# Factors Influencing Post-Transport Behavior, Physiological Responses, and Meat Quality Traits of Japanese Black Cattle

**DOI:** 10.3390/ani15223255

**Published:** 2025-11-10

**Authors:** Gianne Bianca P. Manalo, Jitsuo Mizowaki, Kazunori Mizukami, Makoto Iwamoto, Kenta Koike, Masayuki Nagase, Mitsushi Kobayashi, Shigeru Ninomiya

**Affiliations:** 1 The United Graduate School of Agricultural Science, Gifu University, 1-1 Yanagido, Gifu 501-1193, Gifu Prefecture, Japan; gianne.bianca.pirote.b3@s.gifu-u.ac.jp; 2 JA Hida Meat, 327 Yokamachi, Takayama City 506-0047, Gifu Prefecture, Japan; 3 Faculty of Applied Biological Science, Gifu University, 1-1 Yanagido, Gifu 501-1193, Gifu Prefecture, Japan

**Keywords:** animal welfare, behavior, Japanese black cattle, transportation

## Abstract

**Simple Summary:**

Transportation represents a critical stage in beef production, yet it exposes cattle to multiple stressors that may compromise welfare and carcass quality. This study evaluated 154 Japanese Black cattle to identify transport-related factors influencing welfare outcomes at the slaughter facility. Behavioral responses, cortisol concentrations, and carcass characteristics were measured as indicators of welfare. Heifers, lighter animals, those transported during summer, originating from multiple farms, subjected to higher loading sizes, or lacking previous transport experience, exhibited signs of fatigue and stress. Steers, heavier cattle, and the same farm groups achieved greater carcass weights, whereas those transported under lower loading sizes demonstrated improved marbling and a higher likelihood of attaining premium carcass grades. These results highlight the importance of addressing the cumulative stressors associated with transport. Optimizing transport management can enhance animal welfare, improve meat quality, and contribute to the development of more ethical and sustainable livestock production systems.

**Abstract:**

Adverse effects of transportation arise from the buildup of various stressors, which collectively compromise animal welfare. This study aimed to assess short-term behavioral responses, physiological stress, and meat quality as indicators of welfare in Japanese Black cattle on arrival at the slaughter facility. A total of 154 animals from different production farms were observed. Generalized linear mixed models were used, with fixed effects including animal type, weight, season, source, loading size, distance, transport experience, and their interaction with time periods. Significant post-transport behaviors and elevated cortisol concentration were observed, particularly in heifers, lighter animals, those transported in summer, from multiple farms, at high loading sizes, or without prior transport experience. Steers, heavier animals, and the same farm groups yielded higher carcass weights, while cattle transported under low loading size had improved marbling scores and a higher probability of achieving A5-grade carcasses. These findings suggest that management practices should focus on animals most susceptible to transport stress and strategies such as mitigating heat stress, transporting animals from the same production farm, and reducing loading sizes should be implemented to improve welfare and meat quality upon arrival.

## 1. Introduction

Transportation of live animals is an integral component of livestock production, yet it remains a significant challenge for the industry. It is considered one of the main bottlenecks, affecting animal welfare and health and leading to economic consequences [1]. Despite public concern, this stage of production has received relatively limited attention from policymakers and researchers.

In Japan, Japanese Black cattle play a significant role in both the country’s culture and economy, primarily due to their distinctive beef characteristics and high levels of marbling, making them the most prominent among Japan’s Wagyu breeds [2]. However, their production presents several challenges, including the need for long-distance transportation because production and processing facilities are often geographically distant [3]. According to the National Livestock Breeding Center, Japanese Black cattle are typically transported two to three times during their lifespan. A recent study [4] reported that transport distances can range from approximately 130 km (about 3 h) to over 2600 km (up to 96 h), depending on the locations of farms and destination facilities. 

This phase of production is particularly critical, as transportation can have a substantial impact on animal welfare. Transportation-induced stress has been identified as a major risk factor for the onset of bovine respiratory disease (BRD) [5,6], as it compromises the immune system and makes cattle vulnerable to opportunistic pathogens, resulting in economic losses of approximately 3 million yen annually on a farm in Miyazaki Prefecture [7] and up to one billion USD in the U.S. beef industry [8]. Furthermore, previous studies have investigated various factors influencing cattle welfare during transportation, such as animal type [9,10], weight [11], season [3,12], distance [13,14], source of origin [15,16], loading or stocking density [17,18], and transportation history [19], all of which have been shown to affect transported animals. These findings underscore the complexity of the transportation process and the necessity of optimizing each component to mitigate adverse effects on animal welfare and overall production efficiency. To effectively address these challenges, it is essential to identify and understand the specific factors that contribute to animal stress during transport.

In welfare studies, identifying potential stressors, whether psychological or physical, is crucial for understanding animals’ responses [20]. Animal-based indicators, such as behavioral patterns, physiological measures, and carcass traits, have been widely used to evaluate well-being during pre-slaughter activities [21,22,23,24]. We hypothesize that these welfare indicators will be significantly influenced by transport-related factors during the post-transport period at the slaughter facility.

While previous studies often focused on isolated transport-related factors, the present investigation provides a broader perspective on livestock transportation by evaluating multiple factors alongside several welfare indicators during this critical phase. This study lays the groundwork for a more integrated understanding of animal adaptations to transport. Conducted under commercial conditions, it allows stressors and animal responses to emerge naturally during routine operations, ensuring that the findings are directly applicable to real-world livestock management. Furthermore, public concern over how animals are handled prior to slaughter continues to grow, prompting some producers to adopt more ethical practices to meet consumer demand and gain a competitive market advantage [25]. The findings of this study will provide practical insights for producers, transporters, and slaughterhouse personnel by identifying critical aspects of transit that influence animal welfare and overall production efficiency. This will also serve as a foundational guide for establishing science-based animal transport protocols and improving industry standards.

## 2. Materials and Methods

### 2.1. Animals and Study Design

A total of 154 Japanese Black cattle, with an average age of 28.6 months, were used in this study. Of these, 85 animals were assessed for behavioral observation, and 69 were used for cortisol analysis. Meat quality data were collected from both the behavior and cortisol groups.

Animals were selected using a stratified random sampling approach. Animals were first grouped into strata based on factors, including animal type, season, distance, and source. Within each stratum, animals were randomly selected and then allocated according to weight category, loading size, and transport history, ensuring balanced and representative coverage across all relevant factors.

### 2.2. Transportation Condition

In the slaughter facility where this study was conducted, the design and type of transport trucks were standardized. All vehicles were well-ventilated and equipped with non-slip metal flooring to ensure animal safety during transport. Animals were transported to the JA Hida Meat slaughter facility located in Yokamachi, Takayama City, Gifu Prefecture, Japan, between 9:00 a.m. and 2:00 p.m., from April 2022 to March 2025.

### 2.3. Environment of the Slaughterhouse

At JA Hida Meat’s holding facility, 70 individual stalls were provided, each measuring 80 cm in width and constructed with iron partitions (105 cm height, 120 cm depth). Each stall was equipped with both a feed and a water trough. The floor was bare concrete with no bedding provided. Upon arrival, each selected animal was individually confined to a stall and remained there until the day after arrival, when slaughter took place. During this confinement period, feed (Fusuma A, produced by Nisshin Flour Milling Co., Ltd., Tokyo, Japan) was offered twice, once in the afternoon of arrival and again the following morning at approximately 200 g per feeding. Water was always available through the water tanks.

### 2.4. Categorical Factors

#### 2.4.1. Animal Type

Steers are castrated male cattle commonly used in beef production.

Heifers are female cattle that have not calved.

#### 2.4.2. Weight

Light cattle are those weighing less than 740 kg at the time of transport.

Heavy cattle are those weighing more than 740 kg at the time of transport.

#### 2.4.3. Season

Winter refers to animals transported from December to February.

Spring refers to animals transported from March to May.

Summer refers to animals transported from June to August.

Autumn refers to animals transported from September to November.

#### 2.4.4. Source or Transportation Method

Same farm means that all animals in the transport group originated from a single production farm.

Multiple farms means that animals were collected from different production farms before being transported together to the slaughter facility.

#### 2.4.5. Loading Size

Low loading size refers to transport groups containing five or fewer animals per vehicle.

High loading size refers to transport groups containing more than five animals per vehicle.

#### 2.4.6. Distance

Short distance refers to transport distances of less than 40 km.

Middle distance refers to transport distances between 41 and 80 km.

Far distance refers to transport distances of more than 80 km.

#### 2.4.7. Transportation History

Experienced animals are those that have undergone transport at least once before being moved to the slaughter facility.

Non-experienced animals are those being transported for the first time directly to the slaughter facility, with no prior transport experience.

Information on animal type, body weight, and season of delivery was obtained from the records provided by the slaughter facility. The source or transportation method and loading size were confirmed through the video recordings captured by the facility’s monitoring cameras and verified with data provided by the slaughter facility. Transport distance was estimated using the address information of each farm obtained from the Gifu Prefecture search service and validated through Google Maps. The transportation history of each animal was retrieved from Japan’s National Livestock Breeding Center (NLBC) System using the 10-digit Individual Identification Number (IIN).

### 2.5. Data Collection

#### 2.5.1. Behavior

Table 1 presents the list of behaviors and postures used to assess Japanese Black Cattle (JBC) in this study. Behavioral recording began as soon as the animals were tied and settled in their designated stalls after transportation. Observations were conducted over a 6-hour period using video recordings captured by four fixed cameras directed toward the eight stalls, providing consistently clear visibility from the front-facing angles.

Focal sampling was employed as the sampling rule, wherein a single animal was selected as the focal subject for observation at any given time. For the recording rules, one–zero sampling was applied to document the occurrence or absence of eating and drinking behavior, while instantaneous sampling was used to monitor rumination activities. Both sampling methods were conducted at one-minute intervals. Continuous recording was used to analyze postural behaviors, specifically standing and lying positions.

#### 2.5.2. Saliva Samples

Saliva samples were collected at three time points from selected animals: on arrival, 3 h post-transport, and 6 h post-transport. Each animal chewed on a cotton swab held with forceps for 30 s until the swab was soaked. The samples were immediately refrigerated in the slaughter facility and placed in an ice box for transport to the laboratory, where the swabs were centrifuged for 15 min using a KUBOTA (Osaka, Japan) compact tabletop refrigerated centrifuge (Model 2800) to extract the saliva. The extracted saliva was then stored at −20 °C until analysis.

A high-sensitivity, expanded-range salivary cortisol enzyme immunoassay kit (Salimetrics LLC, State College, PA, USA) was used for cortisol analysis. Assays were performed according to the manufacturer’s instructions. Before analysis, samples were thawed and centrifuged for 10 min at room temperature using a TC-Meteor 7.2 K Mini centrifuge (Topscien Instrument Co., Ltd., Ningbo, China). An orbital shaker (Allsheng Instruments Co., Ltd., Hangzhou, China) was used during the procedure. A Wellwash ELISA microplate washer (Thermo Fisher Scientific Oy, Vantaa, Finland) was used to wash the microplates. Optical density (OD) at 450 nm was measured using a Multiscan FC microplate reader (Thermo Scientific, Waltham, MA, USA). Results were calculated using a four-parameter logistic (4 PL) curve calculator (AAT Bioquest, Inc., Pleasanton, CA, USA).

#### 2.5.3. Meat Quality Parameters

Meat quality parameters including carcass weight, dressing percentage, yield and quality grade, beef marbling and color standard were obtained from the slaughter facility for analysis. Dressing percentage was calculated by dividing carcass weight by live weight and multiplying the result by 100. Meat quality grading followed the standardized guidelines set by the Japan Meat Grading Association. According to these guidelines, grading was performed at the cross-section between the 6th and 7th ribs. The carcass was evaluated for both yield grade and meat quality grade. The yield grade represents the proportion of edible meat obtained from the carcass, with Grade A (≥72%) indicating above-average yield, Grade B (69–72%) representing average yield, and Grade C (≤69%) indicating below-average yield. Quality grade was assigned on a scale from 1 to 5, with 5 representing the highest quality. Marbling was evaluated using the Beef Marbling Standard (BMS), which ranges from 1 to 12; higher values indicate greater intramuscular fat content. Meat color was assessed using the Beef Color Standard, graded from 1 to 7, where higher scores correspond to darker meat color.

### 2.6. Statistical Analysis

Statistical power analysis was conducted using G*Power (version 3.1.9.6) to determine the required sample size for a repeated measures with a within-between interaction design. The analysis was set a priori with the following parameters: medium effect size (f = 0.25), significance level (α = 0.05), and statistical power (1 − β) = 0.80. The results indicated a required total sample size of 54 animals only to achieve an actual power of 0.86. All statistical analyses were performed using R (version 2025.05.1+513). Median weights were calculated to classify the animals into a weight category. Behavioral frequencies were calculated by counting the number of occurrences of each observed behavior within a specified observation period. For postural positions, the duration of each posture (in minutes) was continuously recorded throughout the observation period.

Behavioral data were analyzed using generalized linear mixed models (GLMMs) with a negative binomial distribution to account for overdispersion in count data. For the behaviors, data were reshaped into long format to include repeated measures across two non-overlapping time periods (0–3 h and 3–6 h), while the cumulative period (0–6 h) was analyzed separately. The fixed effects in the models are factors such as animal type, weight, season, source or transportation method, loading size, distance category and transportation history. Interaction of single factor with time points was also included as a fixed factor. The individual animal was treated as a random effect to account for repeated measures within individuals. Pairwise comparisons among significant fixed effects were performed to identify specific group differences. Statistical significance was set at *p* < 0.05. Descriptive statistics (mean, standard deviation, and sample size) were also calculated to support the interpretation of model results.

Due to facility limitations, cortisol samples were only collected from October 2024 to March 2025; therefore, the season factor was excluded from the analysis. Transportation history was also omitted from the cortisol analysis because all sampled animals had previous transport experience. Due to right-skewed distribution (confirmed via histogram and skewness analysis), cortisol values were log-transformed. A generalized linear mixed model (GLMM) was then fitted using log-transformed cortisol as the response variable, with categorical factors, time, and their interaction as fixed effects, and individual animal as a random effect to account for repeated measures. Pairwise comparisons were computed to evaluate adjusted group differences. Additionally, means, standard deviations, and sample sizes were also summarized.

To analyze the effects of categorical factors in meat quality traits, generalized linear models (GLMs) were employed. Carcass weight and dressing percentage, as continuous variables, were modeled using GLMs with a Gaussian distribution and identity link function to estimate group differences in mean values. Beef marbling standard (BMS) and beef color standard (BCS), treated as ordinal variables, were analyzed using Cumulative Link Mixed Models (CLMMs) with a logit link to evaluate the effects of fixed factors. For each factor, a full model was compared with a null model using likelihood ratio tests to assess significance. Statistical significance was set at *p* < 0.05. Model assumptions, including the proportional odds assumption and the appropriateness of the link function, were evaluated to ensure the suitability of the modeling approach.

To assess whether the distribution of the carcass grades (A5 and A4) differs across categories, the analysis first simplifies the carcass grade variable into a binary factor (A5 vs. A4). Chi-square tests are applied to determine if there is a significant association between the factor and the presence of carcass grade. This approach allows for a clear understanding of whether and how categorical factors influence the likelihood of obtaining the highest carcass quality.

## 3. Results

### 3.1. Drinking, Eating, and Rumination

The mean occurrences of behaviors observed at the specified time points across the categorical factors are presented in Table 2.

A significant decrease in drinking behavior was observed in heifers from Period A to Period B (*p* = 0.001). Similarly, eating behavior in heifers declined significantly over the same period (*p* = 0.001). Rumination was significantly higher in heifers than in steers during Period A (*p* = 0.04). In steers, rumination activity was significantly elevated 3 to 6 h post-transport (*p* = 0.0001).

Across weight categories, both light and heavy animals exhibited a significant decline in drinking (*p* = 0.001) and eating (*p* = 0.05) behaviors from Period A to B.

A similar pattern was observed across seasons, with all animals showing a marked drop in drinking behavior after 3 h (*p* = 0.001). Rumination was lowest in summer during Period B (*p* = 0.03) and higher in winter (*p* = 0.03) and autumn (*p* = 0.03) over the entire observation period. Additionally, rumination was significantly higher in Period B compared to Period A in winter (*p* = 0.04) and spring (*p* = 0.03).

Animals from both same-farm and multiple-farm sources exhibited a significant decline in drinking responses from Period A to Period B (*p* = 0.0001). During Period B, multiple-farm animals showed higher drinking (*p* = 0.007) and eating (*p* = 0.006) behavior compared to same-farm animals. Across the entire observation period, multiple-farm animals consistently maintained higher drinking (*p* = 0.05) and eating (*p* = 0.04) activities than those from the same farm.

In terms of loading size, both low and high-loading groups showed a significant decline in drinking behavior from Period A to B (*p* = 0.0001). Animals transported under low loading size (LLS) exhibited higher drinking frequencies during Period A (*p* = 0.02) and overall observation (*p* = 0.02) compared to high loading groups. Eating behavior also decreased (LLS: *p* = 0.04, HLS *p* = 0.05) in both groups after 3 h post-transport.

For transport distance, animals transported over both short and middle distances experienced a decline in drinking responses (*p* = 0.0002) from Period A to B.

Animals with no prior transport experience exhibited significantly higher drinking behavior on arrival or during period A (*p* = 0.01) and overall observation (*p* = 0.03) compared with the animals that had already undergone transport.

### 3.2. Postural Positions

The mean duration (minutes) of postural behaviors for each category is presented in Table 3. Heifers exhibited longer standing durations throughout the observation period (*p* = 0.03), whereas steers spent more time lying during Period A (*p* = 0.01) and over the entire observation (*p* = 0.05). In addition, in Period B, heifers exhibited longer (*p* = 0.027) lying durations than period A.

Lighter animals stood for longer durations than heavier animals throughout the observation period (*p* = 0.028), whereas heavier animals exhibited prolonged lying behavior (*p* = 0.0017). In addition, heavier animals stood longer (*p* = 0.003) in 3 h post-transport (period A) compared to the following hours (period B).

Animals transported during the autumn season exhibited prolonged lying 3–6 h post-transport (Period B; *p* = 0.04) compared with lying durations on arrival (Period A).

### 3.3. Saliva Cortisol Concentration

**Figure 1:**
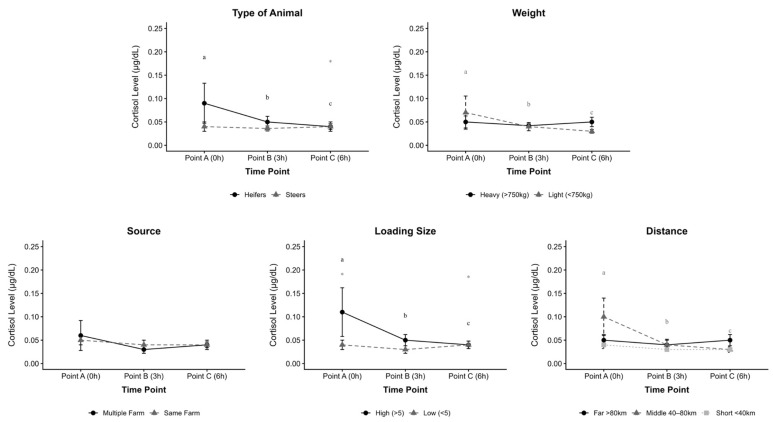
Cortisol concentrations of Japanese Black Cattle (JBC) across different categories and time points. Data represent mean and standard deviation. * for significant values between factors. abc superscript—for significant values within time points.

The mean saliva cortisol concentration of Japanese Black Cattle (JBC) across different categories is summarized in Figure 1. Heifers had higher (*p* = 0.002) cortisol levels on arrival (0 h), which significantly decreased in 3 h and 6 h. A significant interaction was observed between steers and heifers in 6 h (*p* = 0.015), suggesting that steers exhibited a smaller reduction in cortisol upon arrival up to 6 h compared to heifers.

Lighter animals had higher (*p* = 0.02) cortisol concentration on arrival, which dropped sharply between 3 h and 6 h.

There was a significant interaction between loading size groups, with animals in the high loading size group exhibiting significantly higher cortisol concentrations upon arrival (*p* = 0.01) and a greater decline from arrival to 6 h (*p* = 0.01) compared with those in the low loading size group. A significant reduction in cortisol concentration was also observed within 6 h in the high loading size group (*p* = 0.02).

Middle distance group of animals had higher (*p* = 0.03) cortisol levels on arrival (0 h), which significantly decreased in 3 h and 6 h post-transport.

### 3.4. Carcass Parameters

Steers had significantly higher (*p* = 0.0001) carcass weights than heifers (Table 4). Likewise, heavier animals had greater (*p* = 0.0001) carcass weights compared to lighter ones. Animals from multiple farms had lower (*p* = 0.006) carcass weights than those from the same farm.

Loading size significantly influenced beef marbling scores (BMS) (χ^2^ = 8.94, *p* = 0.0028), with animals in low-loading-size groups exhibiting higher BMS compared with high-loading-size groups. Median BMS was 10 in low-loading-size and 9 in high-loading-size groups. A higher proportion of carcasses from the low loading size (LLS: 79) group were graded A5 compared to the high loading size (HLS: 43) group. A statistically significant association was observed between loading size and A5 grading (*p* = 0.05), suggesting that animals in the LLS group were more likely to produce higher-quality carcasses.

## 4. Discussion

Japanese Black cattle transported from production farms to the slaughter facility displayed notable variation in behavioral patterns, physiological responses, and carcass characteristics across different categories.

### 4.1. Behavioral Responses and Cortisol Concentrations

Behavioral patterns of Japanese Black cattle were generally similar across animal type and body weight categories, with the majority of males classified in the heavier-weight group and most heifers in the lighter-weight group. Ishiwata et al. [3] investigated long-distance transport of Japanese Black × Holstein steers in Japan and found that these animals generally lie down frequently during travel. This is comparable with the findings of the present study, as seen in steers and heavier animals that also demonstrated longer lying durations. Heifers exhibited a greater acute stress response, as reflected by higher cortisol concentrations and elevated behavioral activity during the first few hours after arrival, specifically in eating, drinking, and rumination. The observed behavioral responses of heifers and lighter animals indicate a heightened sensitivity to transport stress. This stress may disrupt immune competence [23], resulting in slower adaptation to a novel environment [3,26]. Consequently, elevated cortisol concentration observed in these groups underscores a stronger physiological stress response to transport stressors [27]. This is consistent with the findings of Hulbert et al. [28], who reported that serum cortisol concentrations were higher in female cattle following transportation. This heightened reactivity may partly explain their increased sensitivity and vulnerability to transport [29].

Seasonal differences were observed, with cattle transported in summer exhibiting lower rumination activities. Extreme climatic conditions are among the major stressors animals face during transit [30]. For instance, under heat stress, cattle increase respiration and sweating to dissipate excess heat [31], but high temperature and humidity limit the effectiveness of these cooling mechanisms, resulting in elevated core body temperature and increased cortisol release. Prolonged exposure to heat also weakens immune response and suppresses lymphocyte activity, thereby increasing susceptibility to infections [32]. For these reasons, priority should be given to heat stress management during warmer seasons, as animals transported during this period exhibited altered behavioral responses as seen in this study. In contrast, behavioral responses during colder months remained relatively stable.

Japanese black cattle from multiple farms exhibited higher drinking and eating frequencies between 3 and 6 h post-transport. A recent investigation involving Japanese Black steers at the same slaughter facility was conducted [33], and comparable findings were realized. Transporting animals from multiple farms may increase behavioral responses, possibly due to longer transport durations, additional handling, and commingling with unfamiliar animals during transit. These conditions can lead to increased social tension and agitation, which may intensify fatigue and dehydration. The results of both studies suggest that exposure to multiple farm management systems may negatively influence animal behavior, particularly drinking and eating activities, which could ultimately compromise cattle welfare. This is also consistent with previous reports showing that cattle from truckloads with multiple sources exhibited undesirable welfare outcomes and higher morbidity rates compared to single-source loads [34,35].

Meanwhile, no substantial differences in behavioral and cortisol data were observed among animals transported over different distances. This may be attributed to the relatively short distances between the production farms and slaughter facilities in the present study, which were considerably shorter than those reported in previous research [36,37,38,39]. A transport study involving pre-weaned calves showed pronounced behavioral and physiological stress responses both during and after transport [37]. This difference could be explained by the greater vulnerability of young calves to stressors that influence their biochemical, hormonal, and metabolic status [40]. In contrast, the animals in the present study were older cattle, with an average age of 28.6 months, and were, therefore, more physiologically mature and less sensitive to transport stress. Furthermore, it is important to recognize that duration or distance alone is not the sole contributing factor [41]. Rather, it is the cumulative impact of multiple stressors that builds up over time and ultimately results in compromised welfare.

Space allowance has been highlighted as a key factor, with previous studies identifying it as a more critical determinant of animal welfare than transport distance or duration [42,43]. The cost of cattle transportation is typically calculated based on animal weight and distance traveled; therefore, maximizing stocking density can reduce transport expenses [44]. A limitation of the present study, however, is that information regarding the actual size of the transport vehicles was not available. As a result, stocking density could not be computed, and instead, loading size or the number of animals per vehicle was considered as a factor. Previous studies have shown that limited space negatively affects welfare during transport [45,46,47]. Consistent with these findings, animals transported under higher loading sizes in this study exhibited reduced drinking behavior and elevated cortisol levels on arrival, indicating increased stress. Reduced water intake may reflect stress-induced suppression of normal behaviors, while elevated cortisol confirms activation of the HPA axis.

This study observed that animals with no prior exposure to transport demonstrated higher drinking activity a few hours after arrival compared with experienced animals. The extent of stress that animals experience during transportation may also be shaped by several other influences, such as their prior exposure to handling and transport [48]. Animals with limited or no transport experience are likely to exhibit greater psychological and physiological strain when subjected to transport for the first time. However, the transportation history factor was not included in the cortisol analysis of this study, as all animals sampled had prior transport experience.

### 4.2. Meat Quality

The greater carcass weights observed in steers and heavier animals may be attributed to their higher live weights and lower stress levels. Poveda-Arteaga et al. [49] also reported that heavier animals transported over short distances exhibited the most desirable carcass traits. The lower carcass weight in multiple farm groups may be associated with prolonged and additional procedures experienced during transit by this group of animals. This condition potentially led to dehydration and subsequent body weight loss, as reflected in their increased drinking and eating behavior. Furthermore, this situation was linked to stressful conditions that led to mobilization of their body reserves [50]. Carcass quality parameters, including beef marbling score and overall quality grade, were superior in the low-loading-size animals. Meat quality outcomes have been shown to be affected by loading size in previous studies [51,52]. The higher Beef Marbling Score (BMS) and greater proportion of carcasses graded A5 in low-loading-size groups suggest that this management approach may not only support better animal welfare but also enhance meat quality during transport. However, the observed association should be interpreted with caution, as it may also be affected by underlying farm or management practices.

## 5. Conclusions

Even under optimal transport conditions, animals are exposed to stressors that can compromise their welfare. This study shows that steers, heavier animals, and those with prior transport experience or transported under low loading conditions cope better, whereas animals transported during summer and from multiple farm sources exhibit unfavorable responses. These findings underscore the importance of tailoring transport strategies to animal type, weight, and prior experience, as well as to the management of animals from multiple farm sources, while also mitigating heat stress and optimizing loading density. Implementing such evidence-based and adaptive practices can improve animal welfare, enhance meat quality, and contribute to more sustainable livestock transport systems.

## Figures and Tables

**Table 1:** Ethogram used in this study to observe Japanese Black cattle behavior.

Behaviors	Definition
Drinking	Puts mouth or tongue on the water surface of the tank
Eating	Puts mouth inside the feeder and licking it with their tongue
Ruminating	Regurgitates previously consumed feed and chews it further in a standing and lying position
Postures	
Standing	Supports body with limbs and abdomen not touching the floor
Lying	Places abdomen in the floor and stretch both hind legs

**Table 2:** Mean occurrences of behaviors observed at the specified time points across categorical factors in Japanese Black cattle.

Factors	*n*	Drinking			Eating			Rumination		
		Period A (0 h–3 h)	Period B (3 h–6 h)	Overall (0 h–6 h)	Period A (0 h–3 h)	Period B (3 h–6 h)	Overall (0 h–6 h)	Period A (0 h–3 h)	Period B (3 h–6 h)	Overall (0 h–6 h)
**Type of Animal**										
Steers	45	36.9 ± 22	26 ± 21.5	63.4 ± 40.5	28 ± 18.4	22 ± 20.5	49.7 ± 33.3	7.93 ± 13.4 ^bB^	11.6 ± 26.2 ^a^	19.6 ± 35.3
Heifers	40	43.6 ± 27.7 ^a^	30.6 ± 24.4 ^b^	74.3 ± 46.2	28.9 ± 21.8 ^a^	22.3 ± 20.5 ^b^	51.2 ± 40.3	18.3 ± 24.8 ^A^	19.5 ± 19	37.8 ± 38.1
**Weight**										
Light (<750 kg)	44	44.2 ± 25.1 ^a^	32 ± 23.1 ^b^	76.1 ± 43.2	28.3 ± 20.7 ^a^	21.7 ± 20.5 ^b^	50 ± 38.6	16.4 ± 24.4	15.8 ± 18.3	32.237.7
Heavy (>750 kg)	41	35.6 ± 24.2 ^a^	24.2 ± 22.3 ^b^	60.3 ± 42.5	28.5 ± 19.3 ^a^	22.7 ± 20.5 ^b^	50.9 ± 34.6	8.93 ± 13.5	14.9 ± 28	23.8 ± 37.4
**Season**										
Winter (Dec–Feb)	16	31.9 ± 22.1 ^a^	28.5 ± 24.2 ^b^	60.5 ± 44.6	17.8 ± 13.8	15.5 ± 15.7	33.2 ± 22	15.6 ± 19.4 ^b^	22.1 ± 16.9 ^aA^	37.6 ± 28.8 ^A^
Spring (Mar–May)	27	40.8 ± 30.5 ^a^	29.4 ± 21 ^b^	68.3 ± 48.4	32.3 ± 24	27.2 ± 24.8	59.4 ± 47.6	6.93 ± 13.5 ^b^	15.9 ± 32.5 ^aAB^	22.9 ± 43.2 ^AB^
Summer (Jun–Aug)	22	48.1 ± 23.6 ^a^	33.1 ± 28.5 ^b^	84.3 ± 44	29.5 ± 20.4	22.4 ± 21.5	51.3 ± 32.2	9.23 ± 14.1	6.27 ± 10 ^B^	15.4 ± 21.6 ^B^
Autumn (Sept–Nov)	20	36.6 ± 18.1 ^a^	21 ± 16.5 ^b^	57.8 ± 30.4	30.5 ± 15.3	20.4 ± 14.6	51.2 ± 27.3	22.4 ± 29.3	19 ± 21.9 ^AB^	41.6 ± 44.9 ^A^
**Source**										
Same Farm	38	36.3 ± 124.5 ^a^	21.9 ± 20 ^bB^	57.3 ± 41.2 ^B^	27.4 ± 22.7	18.5 ± 20.4 ^B^	46 ± 42.1 ^B^	12 ± 20.6	19.6 ± 30.2	31.6 ± 42.5
Multiple Farm	47	43.1 ± 125.1 ^a^	33.3 ± 24 ^bA^	77.6 ± 43.3 ^A^	29.2 ± 17.6	25.1 ± 20.1 ^A^	54 ± 31.3 ^A^	13.5 ± 20	11.9 ± 15.3	25.3 ± 33.3
**Loading Size**										
Low (<5)	42	45 ± 24 ^aA^	29.8 ± 23.1 ^b^	76.5 ± 40.1 ^A^	32 ± 21.9 ^a^	25.8 ± 24.1 ^b^	57.5 ± 41	16.3 ± 24.5	14.3 ± 27.1	32.6 ± 37.8
High (>5)	43	35.2 ± 25.1 ^aB^	26.6 ± 22.8 ^b^	60.7 ± 45.4 ^B^	24.9 ± 17.4 ^a^	18.7 ± 15.4 ^b^	43.5 ± 30.5	9.37 ± 14.1	16.3 ± 19	23.7 ± 37.3
**Distance**										
Short <40 km	27	43.2 ± 28.9 ^a^	28.1 ± 27.6 ^b^	71.1 ± 50.8	35 ± 24	27.6 ± 23.7	62.4 ± 45.5	10.1 ± 21.2	13.4 ± 20.2	23.4 ± 37.6
Middle 40–80 km	31	45.1 ± 26.4 ^a^	31.3 ± 22.2 ^b^	76.3 ± 44.7	27.1 ± 17.6	23 ± 19.4	48.7 ± 30.9	15.5 ± 22.8	13.1 ± 13.6	28.6 ± 29.6
Far >80 km	27	31.1 ± 15.4	24.7 ± 18.4	57 ± 31.1	23.3 ± 16.7	15.9 ± 16.5	40.4 ± 29.6	12.5 ± 15.7	19.8 ± 33.3	32.2 ± 45.8
**Transportation History**										
Experienced	60	35.8 ± 24 ^B^	26.8 ± 22	62.9 ± 42.5 ^B^	26.7 ± 18.8	22.1 ± 20.1	48.6 ± 34.1	11 ± 17.2	15.7 ± 25.5	26.8 ± 37.8
Non-experienced	25	50.3 ± 24.5 ^A^	31.6 ± 25	81.9 ± 43.2 ^A^	32.4 ± 22.4	22.2 ± 21.4	54.7 ± 42.2	17 ± 25.9	14.4 ± 17.4	31.4 ± 37.5

abc superscript—for significant values within time points. ABC superscript—for significant values between factors. ± Standard Deviation.

**Table 3:** Mean duration (min) of postural positions across categorical factors in Japanese Black cattle.

Factors	N	Postures					
		Standing (Minutes)			Lying (Minutes)		
		Period A (0 h–3 h)	Period B (3 h–6 h)	Overall (0 h–6 h)	Period A (0 h–3 h)	Period B (3 h–6 h)	Overall (0 h–6 h)
**Type of Animal**							
Steers	45	176.09 ± 11.15	148.42 ± 51.81	325 ± 54.1 ^B^	11.9 ± 38.3 ^A^	19.6 ± 33.2	31.5 ± 49.6 ^A^
Heifers	40	179.1 ± 4.20	169 ± 32.88	348 ± 54.1 ^A^	0.9 ± 4.2 ^bB^	11 ± 32.9 ^a^	11.9 ± 32.9 ^B^
**Weight**							
Light (<750 kg)	44	179 ± 5.34	169 ± 33.2	347 ± 33.7 ^A^	5.46 ± 27.4	7.36 ± 20.7	12.8 ± 33.7 ^B^
Heavy (>750 kg)	41	176 ± 11.2 ^a^	147 ± 52.9 ^b^	323 ± 55.2 ^B^	8.09 ± 29.7	24.3 ± 41.1	32.4 ± 50.5 ^A^
**Season**							
Winter (Dec–Feb)	16	180 ± 11.2	178 ± 3.53	358 ± 3.53	0	1.95 ± 3.53	1.95 ± 3.53
Spring (Mar–May)	27	174 ± 13.5	156 ± 51.2	330 ± 55.3	19.3 ± 48.2	10.8 ± 24.8	30.1 ± 55.3
Summer (Jun–Aug)	22	179 ± 5.18	150 ± 48.8	329 ± 49.2	1.24 ± 5.18	21.9 ± 35.9	23.2 ± 36.7
Autumn (Sept–Nov)	20	179 ± 5.37	154 ± 47.3	333 ± 47.1	1.2 ± 5.37 ^b^	25.8 ± 47.3 ^a^	27 ± 47.1
**Source**							
Same Farm	38	176 ± 11.6	162 ± 39.3	338 ± 43.9	3.64 ± 11.6	17.9 ± 39.3	21.5 ± 43.9
Multiple Farm	47	178 ± 5.34	155 ± 49.1	333 ± 49.1	9.23 ± 36.8	13.6 ± 27.5	22.9 ± 43.6
**Loading Size**							
Low (<5)	42	177 ± 10.4	156 ± 47.3	338 ± 42.8	3.35 ± 10.4	19.6 ± 40.5	21.7 ± 42.8
High (>5)	43	178 ± 6.63	160 ± 42.9	333 ± 50.6	10 ± 38.6	11.6 ± 23.8	22.9 ± 44.7
**Distance**							
Short <40 km	27	179 ± 4.62	163 ± 29.1	342 ± 28.9	1 ± 4.62	14.4 ± 28.8	17.7 ± 28.9
Middle 40–80 km	31	175 ± 12.7	159 ± 49.8	334 ± 53.9	11 ± 33.8	15.4 ± 40.3	26.4 ± 53.9
Far >80 km	27	179 ± 4.68	152 ± 52.3	331 ± 52.7	7.68 ± 34.8	14.4 ± 28.8	22.1 ± 43.4
**Transportation History**							
Experience	60	178 ± 4.88	157 ± 4.88	334 ± 4.78	8.76 ± 33.5	15.1 ± 28.5	23.8 ± 44.1
Non-experienced	25	180 ± 7.49	163 ± 7.49	342 ± 7.49	1.86 ± 5.56	16.6 ± 42.9	18.5 ± 42.7

abc superscript—for significant values within time points. ABC superscript—for significant values between factors. ± Standard Deviation.

**Table 4:** Carcass Characteristics of Japanese Black Cattle (JBC) Across Categorical Factors.

Factors	N	Carcass Weight (kg)	Dressing Percentage (%)
**Type of Animal**			
Steers	89	486 ± 49.2 ^A^	62.7 ± 2.07
Heifers	65	433 ± 38.6 ^B^	63.2 ± 1.74
**Weight**			
Light (<750 kg)	80	424 ± 35.9 ^B^	62.9 ± 1.77
Heavy (>750 kg)	74	505 ± 28.8 ^A^	63 ± 2.13
**Season**			
Winter (Dec–Feb)	53	451 ± 49.5	62.4 ± 2.11
Spring (Mar–May)	39	477 ± 57.8	63.3 ± 1.78
Summer (Jun–Aug)	22	473 ± 47.7	62.8 ± 1.87
Autumn (Sept–Nov)	40	462 ± 49.1	63.4 ± 1.82
**Source**			
Same Farm	70	477 ± 41.6 ^A^	63.2 ± 1.86
Multiple Farm	84	454 ± 56 ^B^	62.8 ± 2
**Loading Size**			
Low (<5)	72	459 ± 51.3	63.2 ± 2.19
High (>5)	82	469 ± 52.8	62.5 ± 1.42
**Distance**			
Short <40 km	54	470 ± 55.3	62.8 ± 1.71
Middle 40–80 km	57	458 ± 47.1	62.7 ± 2.3
Far >80 km	44	461 ± 54.3	63.3 ± 1.7
**Transportation History**			
Experienced	129	465 ± 52.9	62.8 ± 1.93
Non-experienced	25	453 ± 46.4	63.6 ± 1.8

abc superscript—for significant values within time points. ABC superscript—for significant values between factors. ± Standard Deviation.

## Data Availability

Data are available upon reasonable request.

## Abbreviations

The following abbreviations are used in this manuscript:

JBC	Japanese Black Cattle
LLS	Low Loading Size
HLS	High Loading Size
